# An unexpected finding after endoscopic management for dysplastic Barrett’s esophagus

**DOI:** 10.1016/j.igie.2024.09.006

**Published:** 2024-09-28

**Authors:** Rajit Aziz Gilhotra, Angad Walia, Wei Xiong, Roberto Trasolini, Neal Shahidi

**Affiliations:** 1 Department of Medicine, University of British Columbia, Vancouver, BC, Canada; 2 Division of Gastroenterology, St. Paul’s Hospital, Vancouver, BC, Canada; 3 Division of Gastroenterology, Vancouver General Hospital, Vancouver, BC, Canada; 4 Department of Gastroenterology, Royal Brisbane and Women’s Hospital, Brisbane, Australia; 5 Department of Pathology and Laboratory Medicine; St. Paul’s Hospital, Vancouver, BC, Canada

A 78-year-old man with C3M3 Barrett’s esophagus (BE) and previous band mucosectomy for nodular dysplasia was referred for endoscopic submucosal dissection (ESD) of an early esophageal cancer. ESD was successfully performed, and histopathology identified a well-differentiated intramucosal adenocarcinoma without lymphovascular invasion and negative histologic margins. At 3 months, interval endoscopy was performed; the post-ESD scar was identified with re-epithelization, and radiofrequency ablation was performed for residual BE. Subsequent surveillance gastroscopy at 6 months identified extensive fleshy pink polypoid tissue at the earlier ESD site that was not previously present. This finding was atypical for recurrent adenocarcinoma (Fig. 1). Histopathology with hematoxylin and eosin staining revealed squamous proliferation with central fibrovascular cores with no cytologic atypia in keeping with esophageal squamous papillomatosis (ESP) (Fig. 2A). Human papilloma virus involvement was excluded on immunostaining (Fig. 2B).

**Figure 1:**
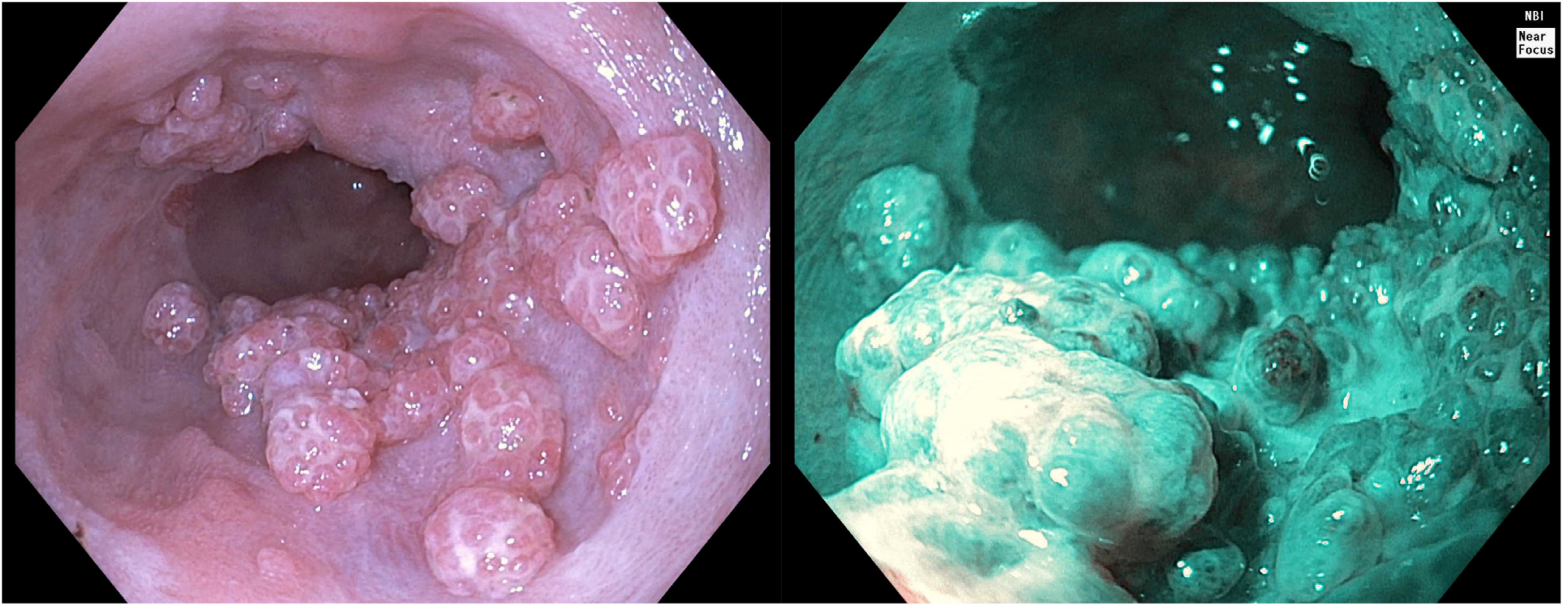
Surveillance gastroscopy 6 months after endoscopic submucosal dissection and radiofrequency ablation.

**Figure 2:**
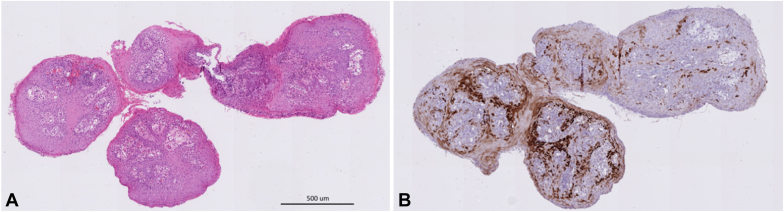
**A,** The hematoxylin and eosin stain shows squamous papilloma with no cytologic atypia (×20). The papilloma is characterized by squamous proliferation with central fibrovascular cores. **B,** The papilloma is not associated with high-risk human papilloma virus and a negative for p16 overexpression by immunostaining (×20).

ESP is a rare and asymptomatic condition that is generally found incidentally at the time of endoscopy; it has an estimated prevalence of 0.01% to 0.45%.^1^ Chronic chemical irritation as well as human papilloma virus infection have been hypothesized to play a role in its pathogenesis.^2^ Historically considered a benign condition, dysplasia and squamous cell carcinoma in resected specimens have been described.^1^, ^2^, ^3^, ^4^ Due to the low risk of malignant progression, in addition to the patient’s age and comorbid disease, he underwent serial endoscopic surveillance; no dysplasia was detected. To our knowledge, this is the first reported case of ESP developing after ESD and radiofrequency ablation, further expanding our knowledge of potential BE endotherapy-related adverse events.

## Patient consent

We have consent from all patients.

## Disclosure

The following authors disclosed financial relationships: R. Trasolini: speaker’s honorarium from Boston Scientific, Medtronic, and Fractyl Health. N. Shahidi: speaker’s honorarium from Pharmascience and Boston Scientific. All other authors disclosed no financial relationships. N. Shahidi is supported by the Michael Smith Health Research BC Scholar Award. There was no influence regarding preparation, review, or approval of the manuscript.
